# All-Inorganic Perovskite Solar Cells: Recent Advancements and Challenges

**DOI:** 10.3390/nano12101651

**Published:** 2022-05-12

**Authors:** Ibrahim M. Maafa

**Affiliations:** Department of Chemical Engineering, College of Engineering, Jazan University, Jazan 45142, Saudi Arabia; imoaafa@jazanu.edu.sa

**Keywords:** inorganic perovskites, power conversion efficiency, operational stability, commercialization

## Abstract

Organic–inorganic metal-halide-based hybrid perovskite solar cells (SCs) have attracted a great deal of attention from researchers around the globe with their certified power conversion efficiencies (PCEs) having now increased to 25.2%. Nevertheless, organic–inorganic hybrid halide perovskite SCs suffer the serious drawback of instability with respect to moisture and heat. However, all-inorganic perovskite SCs have emerged as promising candidates to tackle the thermal instability problem. Since the introduction of all-inorganic perovskite materials to the field of perovskite photovoltaics in 2014, a plethora of research articles has been published focusing on this research topic. The PCE of all-inorganic PSCs has climbed to a record 18.4% and research is underway to enhance this. In this review, I survey the gradual progress of all-inorganic perovskites, their material design, the fabrication of high-quality perovskite films, energetics, major challenges and schemes opening new horizons toward commercialization. Furthermore, techniques to stabilize cubically phased low-bandgap inorganic perovskites are highlighted, as this is an indispensable requirement for stable and highly efficient SCs. In addition, I explain the various energy loss mechanisms at the interface and in the bulk of perovskite and charge-selective layers, and recap previously published reports on the curtailment of charge-carrier recombination losses.

## 1. Introduction

Organic-inorganic metal-halide-based hybrid perovskite solar cells (SCs) have been at center stage since their discovery, mainly due to their remarkable optical and electronic characteristics [1], including high absorption coefficients [2,3,4], higher defect tolerance with low trap density [5,6], longer carrier diffusion lengths and lower exciton binding energies [7,8]. They have recently achievement excellent power conversion efficiencies (PCEs) of 25.2% [9], which places them among the most favorable candidates to open avenues in the field of SCs. The general formula of perovskite material is ABX_3_, where A denotes a monovalent cation (e.g., methylammonium (CH_3_NH_3_^+^(MA^+^)), formamidinium (CH(NH_2_)_2_^+^(FA^+^)), cesium), B is a divalent metal cation (e.g., Pb^2+^, Sn^2+^), and X is a halide (e.g., Cl^−^, I^−^, and Br^−^). It is also possible to place mixed compounds at each site. However, to launch these hybrid perovskite materials onto the market, it is necessary that they are thermodynamically stable and can resist thermal stresses, such as the natural day-night cycle and full sunlight exposure. The organic components, such as MA^+^ or FA^+^, present in perovskites are volatile and cannot withstand much heat, creating a long-term stability issue for the devices while in operation. There are various other environmental factors which can cause the decomposition of hybrid perovskites, such as illumination and humidity, but the major issue of concern is the thermal stability of the perovskite materials in inert atmospheres [10,11,12]. For instance, the decomposition of MA has been reported in the literature at a temperature of 80 °C in MA-based perovskite films [13]. However, photovoltaic devices must be stable at this temperature due to their operating condition requirements. To overcome these limitations in organic–inorganic hybrid perovskite materials, various research groups have attempted to replace organic components with inorganic components, such as cesium (Cs^+^). This has been found to improve the stability of so-called all-inorganic perovskite materials against light, heat, and moisture, ultimately leading to potential pathways toward stable PSCs [11,14,15]. The all-inorganic perovskites are not as photo-sensitive as organic–inorganic perovskites, as a result of which they tend to be more stable under 1 sun continuous illumination. Moreover, the large bandgap of all-inorganic perovskites generates high open-circuit voltages in the solar cells.

It is now recognized that adding a small quantity of cesium can significantly increase the thermal stability of hybrid perovskite materials [16,17]. For instance, FA_0.83_Cs_0.17_Pb(I_0.6_Br_0.4_)_3_ exhibits good thermal and moisture stability even in the open atmosphere. Zeng et al. reported open-circuit voltage $\left(V_{OC}\right)\;\mathrm{values}$ of up to 1.3 V for all-inorganic CsPbI_2_Br and 1.594 V for all-inorganic CsPbBr_3_ [18,19]. However, additional efforts are needed to optimize devices to improve other device parameters. Recently, all-inorganic metal halide perovskites based on cesium, namely CsPbI_3_ [20], CsPbBr_3_ [21,22], CsSn_x_Pb_1−x_I_3−x_Br_x_ [23], CsPb_1−x_Ge_x_I_2_Br [24], Rb_1−x_Cs_x_PbI_3_ [25], and CsPbI_3−x_Br_x_ [26,27], and some lead-free double perovskites Cs_2_AgBiBr_6_ [28,29], CsAgInCl_6_ [30,31], etc., have emerged as thermally stable and efficient candidates for light harvesting. Among the various all-inorganic halide perovskites, CsPbI_3_ perovskite with a cubic phase and band gap (Eg) of 1.73 eV is the most successful. However, it requires a high temperature to preserve its cubic α-phase as it decomposes to a yellow orthorhombic (δ) non-perovskite phase with Eg of 2.82 eV that exhibits inferior photovoltaic properties at ambient temperature and in a humid atmosphere.

An empirical rule is that whether a perovskite material can form a stable cubic lattice depends on an important parameter known as the Gold–Schmidt tolerance factor *t*. This can be written mathematically as *t* = $\left(R_{A}+R_{X}\right)/\surd 2\left(R_{X}+R_{B}\right)$, where $R_{A},\;R_{B}$, and $R_{X}$ are the respective ionic radii [32]. An ideal range for the tolerance factor *t* is close to 1 to produce a stable symmetric cubic lattice. Kim et al. [33] reported that when the tolerance factor decreased below a value of 0.813 or increased beyond 1.107, it led to a major deformation in the cubic ordered lattice. It is noteworthy that the concept of the Gold–Schmidt tolerance factor is based on a hard-sphere model and was primarily conceived for oxide perovskites. However, the tolerance factor alone cannot predict inorganic ABX_3_ perovskite structures. Considering the ions to be hard spheres, Travis et al. [34] concluded that B site cations possessing a radius less than 0.41 could not coordinate in an octahedral fashion without the halide ion being overlapped. Thus, various research groups have employed the octahedral factor $\mu =r_{B}/r_{X}$ to estimate the fitting of the B site cation in the X_6_ octahedral structure [35].

**Figure 1:**
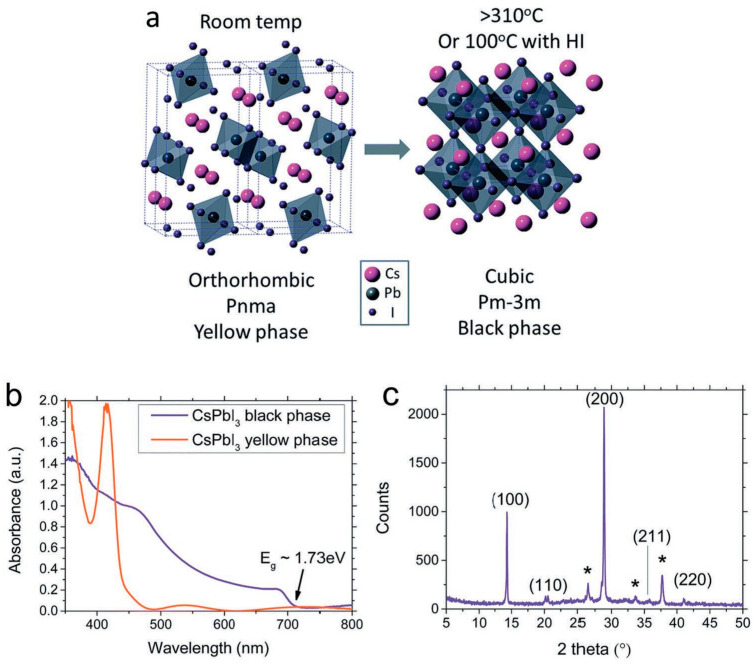
Different phases of CsPbI_3_ all-inorganic perovskite material. (**a**) Lattice structure of orthorhombic yellow (δ) phase and cubic black (α) phase of CsPbI_3_; (**b**) Visible-ultraviolet spectra of yellow and black phased CsPbI_3_ film; (**c**) XRD of black phased CsPbI_3_ film. The peaks correspond to cubic phase lattice having *a* = 6.1769(3) Å. The peaks shown by “*” denote the fluorine-doped tin xxide (FTO) substrate. Adapted with permission from [36].

In addition, due to the octahedral tilt and big macroscopic strains in the crystal, the all-inorganic perovskite becomes thermodynamically unstable with low entropy. However, a more stable phase exists at ambient conditions having minimum Gibbs free energy which is known as the δ-phase, as shown in Figure 1.

## 2. Fabrication of All-Inorganic Perovskite Films

To fabricate high performance all-inorganic PSCs, superior quality perovskite films featuring pinhole-free morphology are important since pinholes can generate a direct means of contact between the selective layers of charge (also called shunts) due to which open-circuit voltage ($V_{OC}$) and overall PCE drop significantly. One of the techniques suggested by Burwig et al. [37] and Zhu et al. [38] that incorporates sublimation of two precursors in a vacuum chamber has produced uniform perovskite films on substrates having large areas. Moreover, this technique does not involve solvents and hence is suited for insoluble or poor-soluble materials, such as bromide precursors.

Ma et al. prepared, for the first time, CsPbIBr_2_ thin films via a dual-source evaporation technique [39]. The films exhibited a bandgap of around 2.05 eV with stable photo luminescence emission at a value of 2.00 eV. The spectrum showed no photo luminescence peaks within the low-energy wavelength range, suggesting the absence of halide segregation in mixed-type halide inorganic perovskite. The HTL-free type planar architecture of CsPbIBr_2_ PSC having the configuration glass/FTO/c-TiO_2_/CsPbIBr_2_/Au exhibited a PCE of 4.7%. Further, Frolova et al. co-evaporated CsI and PbI_2_ precursors to form highly pure and uniform CsPbI_3_ perovskite films having well-aligned crystals [40]. The device attained a highest PCE of 10.5%. Since cesium halides are hygroscopic in nature, to protect the films from ambient air during deposition and measurement processes, Chen at el. employed an all-vacuum-deposition technique [41]. The all-vacuum deposited CsPbI_2_Br SCs exhibited a record PCE of 11.8% showing little hysteresis. Furthermore, this SC exhibited better stability in comparison to organic lead-halide PSCs having similar device configurations. However, it is very difficult to accurately control the stoichiometric ratio of the deposited inorganic perovskite films fabricated by the co-evaporation method because of the difference in evaporation rates of each precursor. Moreover, the rate of evaporation of precursors is affected due to sublimation during co-deposition and creating a vacuum typically requires significant energy consumption in complex equipment settings which can be a demerit for eco-friendly manufacturing.

A better technique to produce high-quality perovskite films has been developed, namely, the solution-chemistry deposition method which is comparatively cheaper and facile. The solution processing method can be broadly classified into two categories: one-step and two-step sequential deposition techniques. The one-step technique involves the deposition of perovskite material films straight from a solution containing all the precursors. However, this technique is not usually employed for preparing inorganic perovskite films as it is widely employed for the preparation of organic–inorganic hybrid perovskite films, mainly because of the low solubility of the inorganic precursors compared to organic precursors. In the case of the two-step technique, lead halide is accumulated on the surface of the substrate, which is followed by the coating of cesium halide on the top to allow the two types of precursors to react to form inorganic perovskites. It should be noted that the characteristics of film produced via solution-processing methods depend substantially on the evaporation and deposition parameters, the properties of solvents, and wetting.

**Figure 2:**
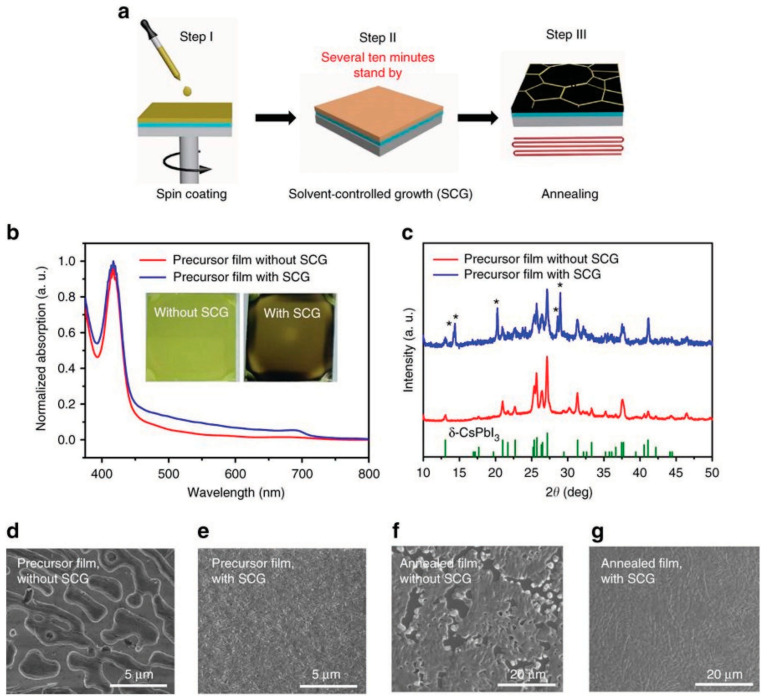
Solvent-controlled growth (SCG) technique for depositing CsPbI_3_ thin film: (**a**) Illustration of film preparation; (**b**) Plots showing normalized absorption spectra of CsPbI_3_ films without and with SCG technique, whereas inset depicts the as-prepared films images without and with SCG; (**c**) X-ray-diffraction plot of the as-prepared CsPbI_3_ film—the peaks dominantly shoot from δ-phase CsPbI_3_ without the SCG technique. A certain amount of the δ- phase CsPbI_3_ has been converted into the β-phase post SCG application. The peaks distinguished with “*” marks originate from β-phase CsPbI_3_; (**d**,**e**) Scanning electron microscope snapshots of the as-prepared CsPbI_3_ films without and with SCG, respectively, and (**f**,**g**) Scanning electron microscope snapshots of annealed CsPb_I3_ films without and with SCG, respectively. Adapted with permission from [42].

To achieve a larger grain size and stable inorganic perovskites, meticulous crystal engineering is required involving precise control of the solution rate. A high quality and stable α-phase CsPbI_3_ film was fabricated by Wang et al. employing the solvent-controlled growth (SCG) of precursor films performed in an ultra-dry atmosphere by retarding the rate of evaporation of the residual dimethyl sulfoxide (DMSO) precursor solvent [42]. A partial phase conversion of CsPbI_3_ from δ- to β- phase was found after SCG processing, suggesting the diffusion of precursors during the evaporation phase of solvent leading to reconstruction of film. This SCG technique yielded annealed pinhole-free CsPbI_3_ films comprising crystals of size larger than 5 μm and delivering PCE of 15.7%, as shown in Figure 2.

A gradient thermal annealing technique was developed by Li et al. that can check the growth of α-CsPbI_2_Br crystals in a green isopropanol antisolvent, which, in turn, can optimize the morphology of the film produced [43]. The α-CsPbI_2_Br crystals can be accurately controlled via this technique producing low-defect-density film with an average grain size of 1 μm. The CsPbI_2_Br film incorporating PSCs attained an efficiency of 16.07%.

**Figure 3:**
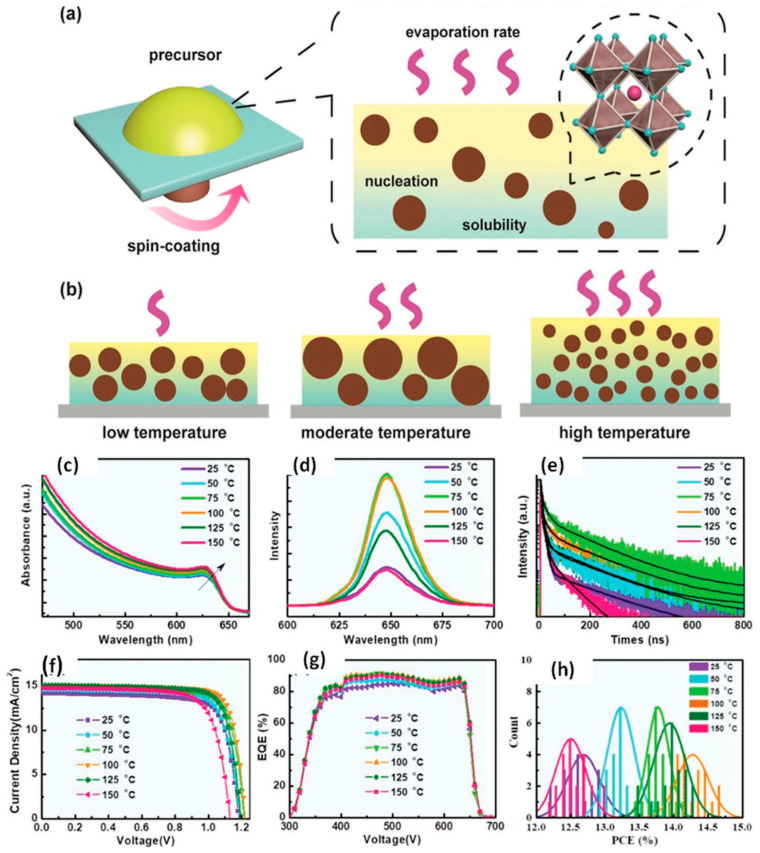
(**a**) Illustration of CsPbI_2_Br thin film growth via spin-coating technique; (**b**) Comparison of CsPbI_2_Br thin film growth at various temperatures; (**c**) Ultraviolet–vis absorption spectra; (**d**) Photo luminescence spectra; (**e**) decay plots; (**f**) *J*-*V* curves; (**g**) EQE spectra; (**h**) efficiency histograms of twenty devices. Adapted with permission from [45].

Yang et al. reported a flash annealing technique falling under the category of a one-step solution process to produce CsPbI_2_Br films having a desired black phase, uniformity, and closely packed highly crystalline morphology [44]. They incorporated SnO_2_ film as an electron transport layer (ETL) which improved the short-circuit current (*J*_SC_) because of comparatively better energy level alignment and higher charge extraction efficiency. The planar architecture PSCs, having a configuration of ITO/SnO_2_/CsPbI_2_Br/2,2′,7,7′-tetrakis(*N*,*N*-di-*p*-methoxyphenylamine)-9,9′-spirobifluorene(Spiro-OMeTAD)/Ag, attained a conversion efficiency of up to 13.09%. It should be noted that the mesoporous layer was removed which made the device cheaper to produce with a high-throughput roll-to-roll printing technique. Since the nucleation process depends substantially on the solubility of the precursors in the solvents at high temperatures, the crystallization from precursor solutions significantly depends on the temperature. It has been shown that nucleation can easily be prevented in a high precursor solubility system with grain growth being controlled by the solvent evaporation. Liu et al. worked with the precursor solution at a temperature of 100 °C to subdue formation of numerous nuclei and to boost the crystallization rate [45]. Consequently, they achieved pinhole-free, homogeneous, large grain size crystals and a thick CsPbI_2_Br film having a PCE of 14.81%, as depicted in Figure 3. This figure presents a comparison of the film growth mechanisms of CsPbI_2_Br film at various temperatures.

In order to produce homogeneous and miniaturized layers to improve the reproducibility of performance characteristics of the device, various researchers have proposed a two-step sequential deposition method. In this direction, most of the researchers synthesized all-inorganic CsPbBr_3_ by depositing the first layer of PbBr_2_ via spin-coating followed by the addition of CsBr through solution-processing and finally post-annealing it. Table 1 summarizes the reported performance characteristics from some research groups that synthesized CsPbBr_3_ via a two-step solution-processing technique.

**Figure 4:**
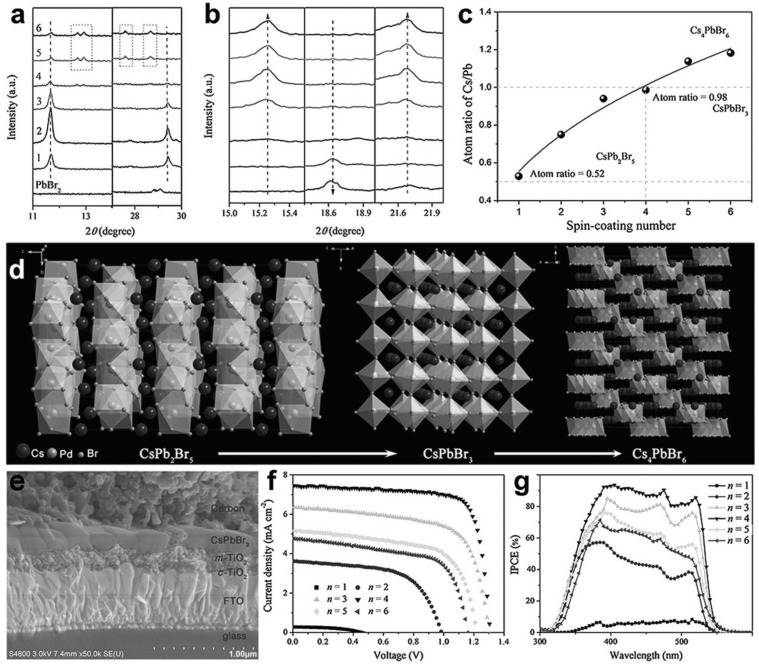
Phase transition of thin films of cesium lead bromide: (**a**) X-ray-diffraction patterns at *n* = 1 to *n* = 6 (where “*n*” denotes the number of CsBr layer deposition cycles) in the 2θ range of 11–30.8°; (**b**) X-ray-diffraction patterns at *n* = 1 to *n* = 6 in the 2θ range of 15° to 22.8°; (**c**) Atomic ratio of Cs/Pb at various cycles of deposition; (**d**) Crystalline architecture of the cesium lead bromide perovskites; (**e**) Scanning electron microscope snapshot of the cross-section of cesium lead bromide perovskite solar cell; (**f**) *J*-*V* characteristics of the device fabricated at various deposition cycles and reported at a standard solar illumination of AM 1.5 G, (**g**) External quantum efficiency (EQE) of the device. Adapted with permission from [48].

Yu et al. first deposited lead halide on the surface of the substrate followed by the deposition of CsBr film in a methanol solution [56]. Dimethylsulfoxide (DMSO) in various quantities was used to ameliorate the CsBr solubility. Consequently, the morphology of the film was highly ameliorated with a reported efficiency of 13.27% and a stable power output (SPO) of 12.5%. However, the drawback of the two-step sequential deposition technique is that the CsBr precursor cannot completely penetrate the pre-deposited lead halide film to accomplish a complete reaction, and hence the efficiency of the SCs decreases. To avoid this problem, Duan et al. proposed a multistep solution-processing technique to fabricate extremely pure CsPbBr_3_ films [48]. They adjusted the number of deposition cycles of CsBr solution to gradually convert CsPb_2_Br_5_ to CsPbBr_3_ to Cs_4_PbBr_6_, and attained monolayer and vertically aligned grains, as shown in Figure 4.

## 3. Compositional Engineering of All-Inorganic Perovskites

Organic–inorganic hybrid perovskite material ABX_3_ is composed of organic cations as A, Pb^2+^ or other divalent metal cation as B, and halide, or a mixture of halides, as X. When the organic cations are replaced by inorganic components, such as cesium (Cs^+^), it leads to the formation of all-inorganic perovskites. The composition of all-inorganic perovskite materials can be engineered under two categories, namely, lead-based and lead-free. These subcategories are discussed in the following subsections.

### 3.1. Lead-Based All-Inorganic Perovskite Solar Cells

The most extensively investigated all-inorganic perovskite material is cubic α-CsPbI_3_ having a 1.73 eV bandgap, which conforms favorably to the solar spectrum. However, this high temperature (α) phase is unstable at ambient temperature, due to which the photovoltaic performance of CsPbI_3_ is gravely hampered. To overcome this problem, various techniques have been proposed by researchers. Eperon et al. synthesized stabilized α-CsPbI_3_ in a vacuum [36]. They added a small quantity of HI to the perovskite precursor and found that it drastically reduced the processing temperature down to 100 °C because of the development of strain in the lattice, ultimately engendering crystal phase transitions. The stability arose through the formation of tiny black phase α-CsPbI_3_ crystals after adding HI. This novel synthesis of a stable α-CsPbI_3_ phase perovskite has opened new avenues for the rapid development of all-inorganic PSCs, although the PCE was limited to only 2.9%. Table 2 summarizes the gradual advancement of the performance characteristics of lead-based all-inorganic PSCs employing various additives to stabilize the perovskite cubic phase.

**Figure 5:**
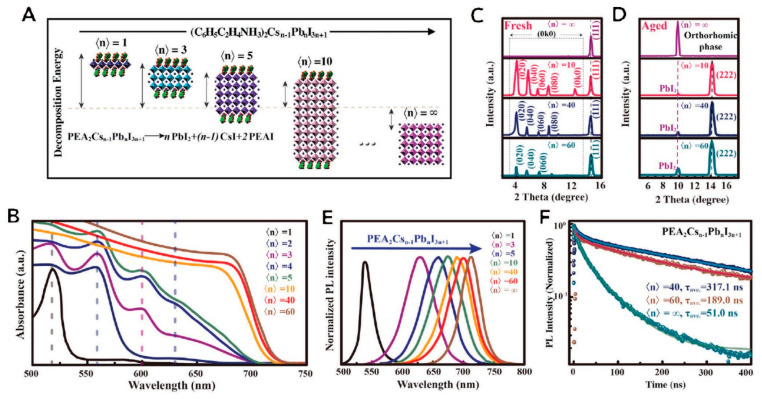
Properties of quasi-two-dimensional all-inorganic perovskite material thin film: (**A**) Unit structure of cell of PEA_2_Cs*_n_*_−__1_Pb*_n_*X_3*n*+1_ and its decompositional energetics employing first principles of density functional theory simulation having various values of n; (**B**) Ultraviolet–vis absorbance spectra having various values of n; (**C**) Low diffraction angle zone of XRD spectra of powdered perovskite; (**D**) XRD pattern of a chosen zone after aging of perovskite thin films; (**E**) normalized PL spectra, and (**F**) Time-resolved PL decay. Adapted with permission from [60].

Jiang et al. synthesized low-dimensional perovskites with adjustable bandgaps by adding phenylethylammonium iodide (PEAI) to α-CsPbI_3_ [60]. The produced quasi-two-dimensional perovskites, having values of *n* = 1, 2 and 3, with chemical formula PEA_2_Cs*_n_*_−1_Pb*_n_*X_3*n*+1_, drastically curbed the unwanted phase transition, thereby lowering the trap density. Density functional theory (DFT) simulations suggested that the quasi-2D perovskites had high decomposition energy. Figure 5 displays the corresponding PL spectra showing a blue-shift in emission with decrease in n values. The reported PCE of these quasi-2D α-CsPbI_3_ perovskite SCs was 12.4% with highly improved stability.

**Figure 6:**
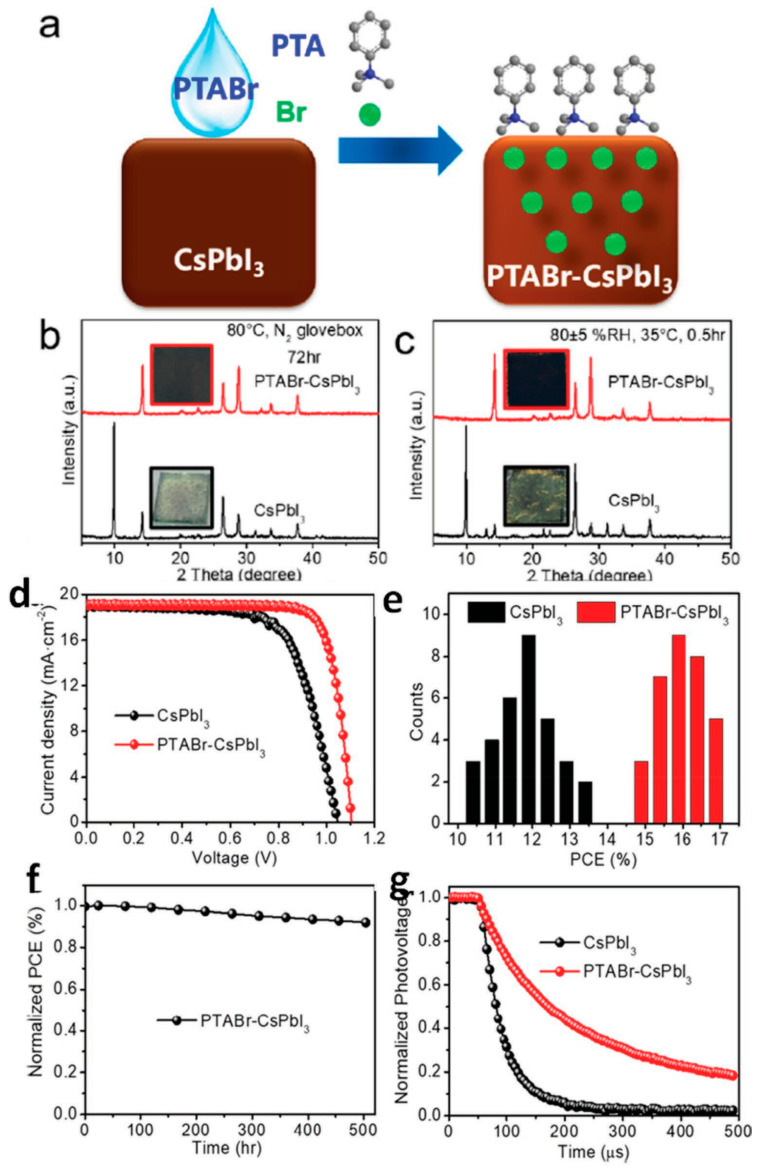
(**a**) Demonstration of gradient-doping of Br and surface passivation of PTA cation on thin film of CsPbI_3_; (**b**) X-ray diffraction (XRD) pattern of PTABr–CsPbI_3_ and CsPbI_3_ perovskite thin film heated at 80 °C in a glovebox filled with N_2_ for a duration of 72 h; (**c**) XRD pattern of CsPbI_3_ and PTABr–CsPbI_3_ perovskite thin film post exposure to a relative humidity (RH) of 80 ± 5% at ≈35 °C for a duration of 0.5 h; (**d**) *J*-*V* curves of CsPbI_3_ and PTABr–CsPbI_3_ based devices under AM 1.5 G simulated illumination condition of 100 mW cm^−2^ during backward scan; (**e**) Histogram of PCE of CsPbI_3_- and PTABr–CsPbI_3_-based devices; (**f**) Normalized PCE of PTABr–CsPbI_3_; and (**g**) normalized output voltage of CsPbI_3_- and PTABr–CsPbI_3_-based devices. Adapted with permission from [58].

Zhao et al. added a small quantity of EDAPbI_4_ perovskite with an EDA cation to obtain stable α-CsPbI_3_ perovskite without the formation of a non-perovskite δ phase [57]. The EDAPbI4 perovskite aided the synthesis of α-CsPbI_3_ perovskite films which were found to be stable at room temperature for many months and greater than 150 h at 100 °C. The fabricated PSCs exhibited a reproducible PCE of 11.8%. In this way, Zhao et al. established that bication molecules were potential candidates to fabricate PSCs with high PCE and stability [57]. Zhao et al. further discovered that phenyltrimethyl-ammonium bromide (PTABr) can be exploited to work as an additive, performing the dual functions of surface ligand passivation and gradient-wise Br-doping for post-treatment of CsPbI_3_ [58]. The above technique produced a pin-hole free morphology of film exhibiting a small increment in the mean grain size of the crystal to approximately 510 nm; however, it exhibited a small blueshift of 5 nm length post $1\;{\mathrm{mg}\;\mathrm{mL}}^{-1}\;$PTABr treatment, as shown in Figure 6. The gradient-wise Br-doping and the addition of an organic PTA cation to the surface drastically improved the moisture resistance and stability of CsPbI_3_. The CsPbI_3_ all-inorganic PSCs treated with PTABr displayed an excellent PCE of 17.06% and an SPO of 16.3%.

**Figure 7:**
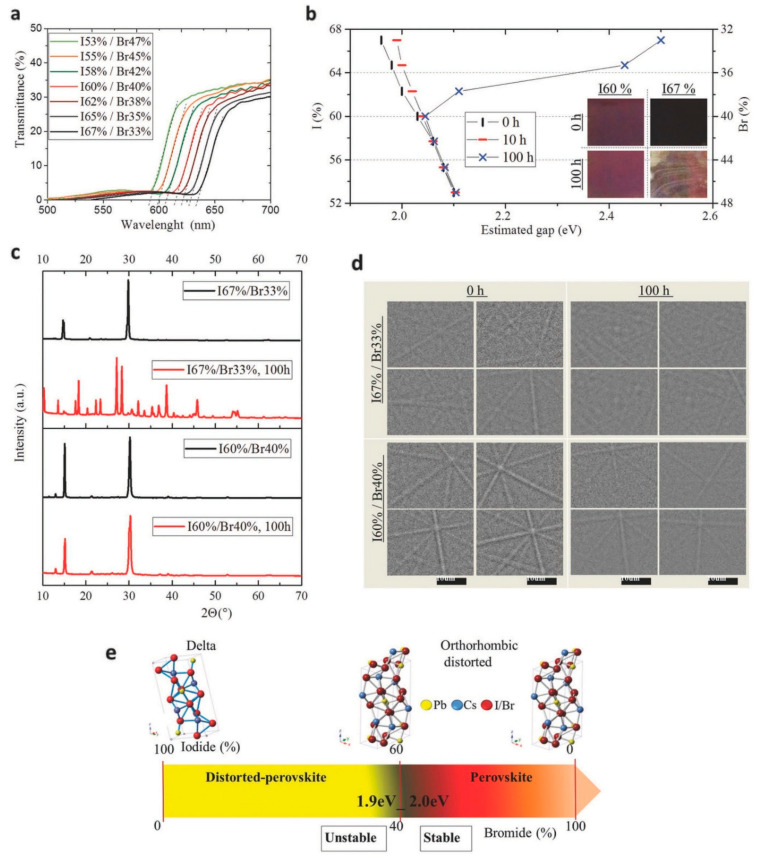
(**a**) Optical transmittance spectra of cesium lead-halide inorganic perovskite thin films at various I/Br ratios; (**b**) Estimated bandgap calculated from the intersection of the wavelength axis (dashed lines) with short-wavelength decay of the transmittance plot. Inset displays the images of freshly synthesized perovskite films and post storage for 100 h duration under ambient conditions; (**c**) X-ray diffraction patterns of films having two I/Br ratios for freshly synthesized and stored samples for 100 h duration in the dark at a relative humidity of 30% at 25 °C; (**d**) Kikuchi patterns that originate due to backscattering diffraction of the perovskite film—data collected at 0 and 100 h storing in the dark at a relative humidity of 30% at 25 °C; and (**e**) Evolution of the crystalline structure of the perovskite with I/Br ratio. Adapted with permission from [75].

The most employed halogens in all-inorganic perovskite materials are I and Br; however, researchers have also utilized pseudohalogens, such as SCN^−^ [73,74]. In addition, researchers have performed compositional engineering on the octahedral halide PbX_6_ sublattice of CsPbX_3_ that permitted bandgap adjustment throughout the whole compositional range from a narrow bandgap CsPbI_3_ to a wide bandgap CsPbBr_3_. The complete replacement of I with Br in CsPbI_3_ dramatically enhanced the thermal stability and elevated the bandgap up to 2.3 eV. However, due to mismatch with the solar spectrum, the device generated low *J*_SC_. In order to overcome the issue of large bandgap formation so as to maximize the light absorption and enhance the thermal stability simultaneously, several groups have explored the tuning of the ratio of iodine and bromine in mixed halide all-inorganic CsPb(I_x_Br_1−x_)_3_ perovskites. In this direction, Sanchez et al. attempted to tune the I/Br ratio and established that, to stabilize the inorganic perovskite black phase at room temperature, the iodine content must be lower than 60% [75]. Specifically, they showed that their compositional engineering rule can produce all-inorganic perovskite SCs that can preserve 90% of their PCE even after exposure to heat at 200 °C for 1 h, as shown in Figure 7.

**Figure 8:**
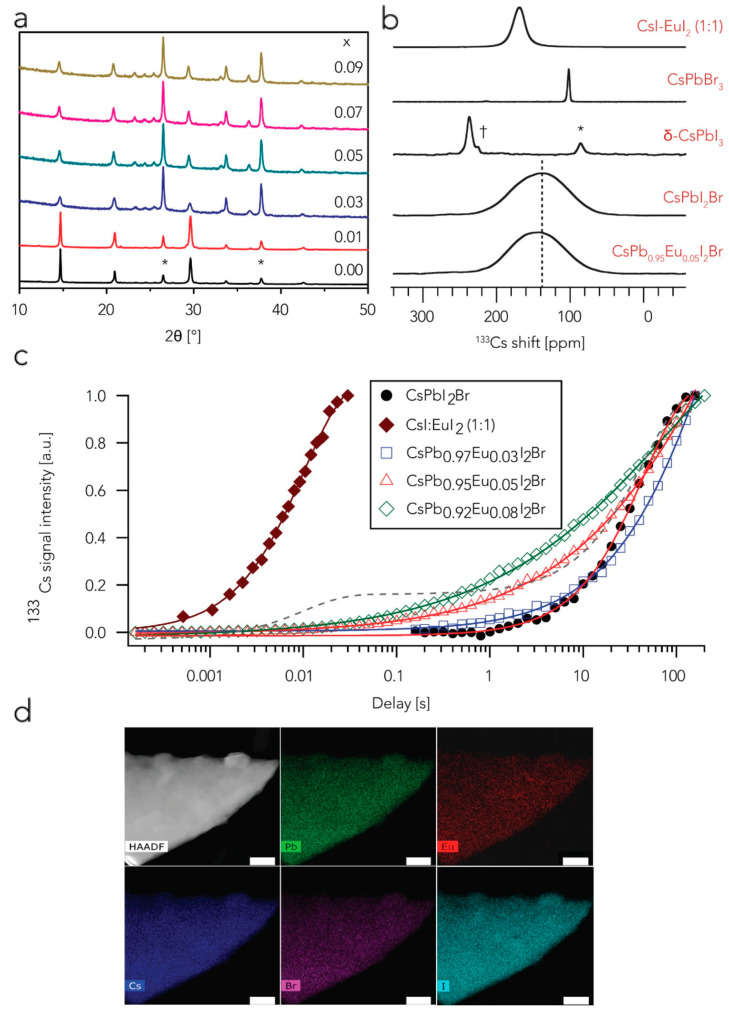
(**a**) XRD patterns of europium-doped CsPb_1__−*x*_Eu*_x_*I_2_Br (0 ≤ *x* ≤ 0.09) perovskite thin films, where * denotes the peaks of fluorine-doped tin oxide substrate; (**b**) ^133^Cs solid state magic angle spinning (MAS) NMR spectra obtained at 11.7 T 20 kHz and temperature of 298 K; † denotes a transmitter artifact while * denotes a spinning sideband; (**c**) Relaxation times of ^133^Cs spin-lattice for various compositions of perovskites—the plots in solid are exponential fits while dashed line represents best biexponential fit of CsPb_0.95_Eu_0.05_I_2_Br perovskite data; (**d**) High-angle annular dark-field scanning transmission electron microscopy (HAADF-STEM) image and individual maps of elements of CsPb_0.95_Eu_0.05_I_2_Br perovskite. The white bar represents 100 nm of scale. Adapted with permission from [67].

Researchers also considered germanium as an alternate option to lead for producing perovskite materials. To this end, Yang et al. synthesized novel inorganic CsPb_1__−*x*_Ge*_x_*I_2_Br perovskite materials under a humid ambience without employing a glovebox [24]. Recently, Xiang et al. demonstrated the synthesis of europium-doped CsPbI_2_Br inorganic perovskite material [67]. They reported that with an optimum doping of europium as in CsPb_0.95_Eu_0.05_I_2_Br, the highest PCE attained was 13.71% with an SPO of 13.35% (Figure 8).

### 3.2. Lead-Free All-Inorganic PSCs

#### 3.2.1. Sn-Based Inorganic PSCs

The toxic and carcinogenic nature of lead has prompted researchers to find a surrogate in large-scale PSC applications since it can pose a serious threat to humans and to the environment. Although a small quantity of lead is employed in the fabrication, there is a high risk of leakage from discarded units [5,76]. Ideally, Sn is the best replacement for Pb in perovskite materials since both exhibit s^2^ valence configuration. However, it has the limitation of being unstable since it can rapidly oxidize on air exposure. To overcome this issue, these type of all-inorganic perovskite devices are required to be fabricated in an inert ambience with a meticulous sheathing arrangement. Moreover, another challenge lies in fabricating uniform and completely sheathed Sn-based perovskite films without employing additives.

A lot of attention has been paid by researchers towards CsSnI_3_ lead-free all-inorganic PSCs because of their lower bandgap of 1.3 eV which is suitable for single-junction SCs. Various advancements have been made in the areas of optimizing the film deposition methods [77], compositional engineering [78,79], and the incorporation of additives in the synthesis of CsSnX_3_ perovskites [80,81,82].

Marshall et al. demonstrated, for the first time, the solution processing of CsSnI_3_ films at room temperature [83]. The synthesized film showed low defect densities; however, the stability of the fabricated SCs was comparatively poor with an efficiency reduction of 30% after storing in the dark and open sunlight for a duration of 1 h. To circumvent this issue, the authors incorporated SnX_2_ (X = F, Cl, Br, and I) as additives [84], since these additives showed propitious performance in resistance against oxidation [85,86]. Amongst these additives, SnCl_2_ showed prominent benefits. It was deposited as a very thin layer over perovskite film, thereby behaving as an effective drying agent for the CsSnI_3_ cubic structure. Consequently, the fabricated unencapsulated CsSnI_3_ device, without a hole transport layer (HTL), exhibited a boost in stability by a minimum of one order of magnitude relative to its lead-based equivalent under the condition of solar-simulated illumination at 50 °C in ambient atmosphere.

**Figure 9:**
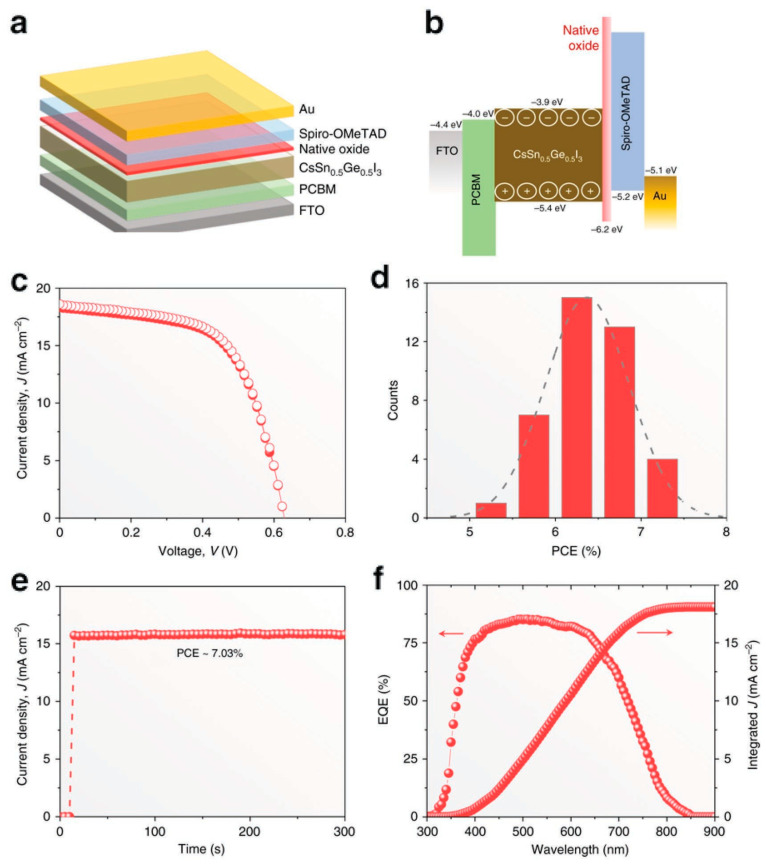
(**a**) Schematic of planar architecture of CsSn_0.5_Ge_0.5_I_3_ thin-film PSC; (**b**) Diagram of its energy level; (**c**) *J*-*V* characteristics; (**d**) Efficiency histogram; (**e**) Stable power output (SPO), and (**f**) EQE spectrum of the device. Adapted with permission from [79].

Chen et al. demonstrated the employment of lead-free, cesium tin germanium triiodide (CsSn_0.5_Ge_0.5_I_3_) all-inorganic perovskite material in the fabrication of perovskite solar cells [79]. They found that a native-oxide Ge (IV) layer of approximately 5 nm thickness was formed rapidly within a duration of 30 s when the film was kept in atmospheric air. This native oxide layer passivated and encapsulated perovskite film and reduced the trap densities of electrons and holes to 1016 ${\mathrm{cm}}^{-3}$. The device exhibited a PCE of 7.11% and high stability with an efficiency decay of less than 10% even after 500 h of prolonged performance in N_2_ ambience under 1 sun illumination, as shown in Figure 9.

#### 3.2.2. Double Perovskite SCs

The substitution of lead by divalent cations falling outside group-IV is not so effective. One can explore the compositional engineering of the perovskite lattice by substituting two Pb^2+^ ions with a monovalent M^+^ and a trivalent M^3+^ ion, resulting in the formation of an A_2_M^+^M^3+^X_6_ 3D double-perovskite structure, also called elpasolite. These double-perovskites comprise of vacancy-ordered (such as Cs_2_TiBr_6_, Cs_2_SnI_6_) and cation-ordered (such as Cs_2_AgBiBr_6_) type structures. More than 350 elpasolites have already been synthesized [87]; however, first-principle calculations suggest that only 11 materials have suitable bandgaps for photovoltaic applications [88]. These perovskite materials offer better phase stability, lower exciton-binding energy, lower mass of the charge carrier and a broad range of tunable optoelectronic characteristics. The feasible options for the M^3+^ cation include Bi^3+^ and Sb^3+^, while M^+^ can be monovalent size-matched cations. Since Bi^3+^ and Sb^3+^ ions have smaller size than Pb^2+^, small inorganic cations at site A can be used. Hitherto, only a few reports are available in the literature about the synthesis of double-perovskite SCs due to various difficulties in the processing of films [89]. The various double-perovskites developed, along with their performance characteristics, are summarized in Table 3.

**Figure 10:**
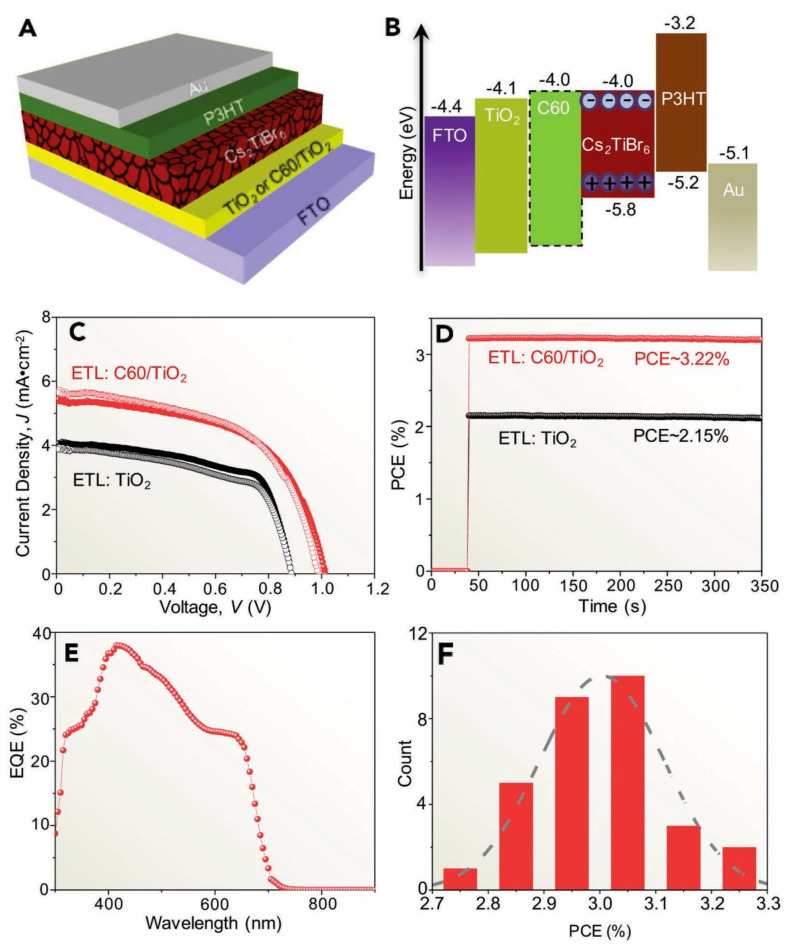
(**A**) Planar architecture of Cs_2_TiBr_6_ perovskite thin film; (**B**) Energy level diagram; (**C**) *J*-*V* characteristics during forward (hollow circles) and reverse (solid circles) scans with and without C60 interfacial layer; (**D**) Stabilized PCE output with and without C60 interfacial layer; (**E**) EQE spectrum with C60 interfacial layer; and (**F**) Efficiency histogram with C60 interfacial layer. Adapted with permission from [93].

Chen et al., for the first time, synthesized high-quality Ti-based thin films of Cs_2_TiBr_6_ double-perovskite for photovoltaic applications using a low-temperature vapor-based technique [93]. The films showed respectable bandgap of 1.8 eV, propitious energy levels, higher stability, long carrier-diffusion lengths beyond 100 nm, and PCEs of up to 3.3%. The device was found to be highly stable against thermal stress, moisture and light, as evident from the characteristics detailed in Figure 10. They further demonstrated that the bandgap of Cs_2_TiI*_x_*Br_6__−*x*_ mixed-halide double-perovskite can be easily adjusted from 1.38 eV to 1.78 eV for both tandem and single junction SCs [95]. Thus, further investigation of these Ti-based double-perovskite materials could open a new avenue in the field of the development of stable and eco-friendly SCs.

Hitherto, Cs_2_AgBiBr_6_ double-perovskite has attracted most attention by researchers due to the presence of a 1.95 eV indirect bandgap within the visible spectrum range. This is an appropriate condition to couple it with a Si light absorber in a tandem SC. Moreover, Cs_2_AgBiBr_6_ exhibits the capacity for defect-tolerance because of the similar electronic characteristics of Bi^3+^ and Pb^2+^. Cs_2_AgBiBr_6_ double-perovskite film was first synthesized by Wu et al. using a low-pressure-assisted solution-processing technique under atmospheric conditions [29]. The fabricated SC exhibited an efficiency of 1.44%. Since these SCs are highly stable under atmospheric conditions without encapsulation of the film, their development can provide a pathway for potential lead-free photovoltaic applications. Furthermore, the bandgap of Cs_2_AgBiBr_6_ can be precisely tuned by doping it with other elements, such as Sb (III) and In (III) [96].

## 4. Flexible All-Inorganic Perovskite SCs

Flexible SCs have witnessed a tremendous growth in recent years because of their propitious characteristics for fabricating portable devices, such as flexibility, compactness and light weight. These portable devices comprise of wearable electronic devices and shaped electronic displays and are presently experiencing unprecedented demand. Moreover, these flexible SCs can be cheaply produced in mass quantities by employing a continuous roll-to-roll technique. This is very advantageous in comparison to the costly and low-manufacturing-speed batch-to-batch fabrication of devices. Hitherto, the highest attained PCE of organic–inorganic hybrid flexible perovskite solar cells is 18.4% [97].

Generally, the synthesis of superior characteristic CsPbI_2_Br films requires annealing at a temperature higher than 250 °C which creates a constraint in working with plastic substrates. To circumvent this problem, Jiang et al. introduced Lewis-base adducts of PbBr_2_ (DMSO) and PbI_2_ (DMSO), which decreased the energy required to form CsPbI_2_Br film and boosted the growth mechanism at low temperature [14]. The fabricated flexible CsPbI_2_Br PSCs generated a PCE of up to 11.73%. The mechanical bending tests of these perovskite flexible films did not show any remarkable PCE deterioration while bending at a radius of curvature of 12 mm for 300 cycles.

Rao et al. employed DMSO to perform solvent annealing at room temperature to obtain CsPbX_3_ flexible perovskites [98]. In this way they were able to control the crystallization dynamics of perovskite, thereby producing uniform films. They fabricated the device with a configuration of polyethylene terephthalate (PET)/ITO/NiO*_x_*/CsPbI_2_Br/C60/bathocuproine/Ag by employing DMSO solvent annealing and thermal annealing at 120 °C. The device exhibited a PCE of up to 7.3%, with a $J_{sc}\;$ of 11.5 mA cm^−2^, $V_{OC}\;$ of 0.97 V, and an FF of of 65.0%. The bending tests showed that the device performance did not degrade even after 100 bending cycles at a 10 mm bending radius.

**Figure 11:**
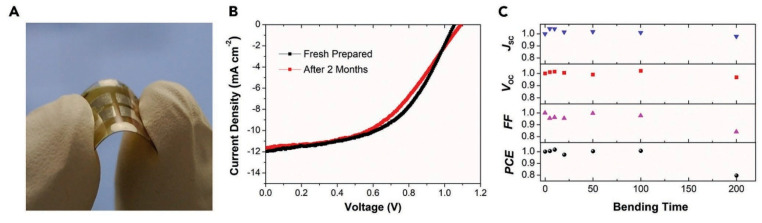
(**A**) An image of a flexible CsPbI_2_Br-based PSC; (**B**) *J*-*V* characteristics of the champion device shown freshly prepared and after two months storage without encapsulation; (**C**) Normalized performance parameters of the solar cell after stressing under various bending cycles at a curvature radius of 4.05 mm. Adapted with permission from [99].

Moving in the same direction to process inorganic CsPbI_2_Br-based flexible PSCs at room temperature, Liu et al. introduced another solvent, 1-methyl-2-pyrrolidone (NMP) [99]. The weak coordination affinity and high solubility of NMP towards cesium lead halide precursor solutions favor the processing of the films at low temperature. Liu et al. synthesized the CsPbI_2_Br films using a vacuum-assisted technique at room temperature. Their flexible inorganic perovskite solar cells fabricated on ITO/PET substrate exhibited an efficiency of 6.50%. The flexible encapsulated solar cells retained a PCE of 6.05% even after storing for a duration of two months in an inert ambience. The mechanical bending tests revealed practically no deterioration in efficiency during the first 100 bending cycles at a radius of curvature of 4.05 mm. However, the efficiency decreased to 80% after bending beyond 200 cycles due to the development of cracks in the flexible ITO/PET substrate. All these characteristics are illustrated in Figure 11.

## 5. Towards Commercialization of All-Inorganic PSCs

The major challenge in front of researchers working in the domain of all-inorganic perovskite SCs is upscaling of these cells [100,101]. Various research groups and firms have fabricated organic–inorganic hybrid perovskite SCs on flexible substrates employing different techniques, including inkjet printing [102,103], slot die printing [104,105], doctor blading [106,107], vacuum evaporation [108], roll-to-roll printing [109,110], screen printing [111,112], and spray coating [113,114], to achieve high stability and cheaper production costs. Hitherto, very few research reports have been published addressing the issue of the upscaling of all-inorganic PSCs. Moreover, the complete transfer of upscaling methods that are employed for organic–inorganic PSCs to fabricate all-inorganic PSCs has been limited in terms of large area film deposition with low defect densities and full coverage. A necessary step towards upscaling on large area substrates with high quality films will be a thorough understanding of the crystallization dynamics and nucleation mechanism in inorganic perovskite film growth. Moreover, the deposition of inorganic perovskite film on both flexible and rigid substrates must be investigated individually, since nucleation and crystallization dynamics for both are different. Although very few findings are available for inorganic perovskite solar cells having an active area of 1 ${\mathrm{cm}}^{2}$ or larger, the results obtained are propitious for upscaling the production of all-inorganic PSCs.

Lei et al. employed dual-source co-evaporation of PbBr_2_ and CsBr precursors to synthesize CsPbBr_3_ perovskite films [108]. The substrate and annealing temperature, and the ratio of the evaporation rate of CsBr to PbBr_2_ was observed to affect the crystallization dynamics and stoichiometry of the grown CsPbBr_3_ films. The grain size was found to be 640 nm and the deposited film was smooth. The planar architecture CsPbBr_3_ PSCs exhibited a high PCE of 5.37% for 1 ${\mathrm{cm}}^{2}$ area substrates.

Liu et al. synthesized pinhole-free and homogeneous CsPbBr_3_ perovskite films by employing TiO_2_/SnO_2_ bilayer electron transport layers (ETLs) and reported a $V_{OC}\;$ of 1.396 V, $J_{sc}\;$ of 6.93 mA, FF of 0.713, and an improved PCE of 6.90% on a 1 ${\mathrm{cm}}^{2}\;$ active area [115]. The improvement in PCE can be linked to better charge carrier mobility in the ETL bilayer and high-quality deposited film. Specifically, the CsPbBr_3_ SCs without encapsulation showed no degradation in efficiency even after storing for over 1000 h in surrounding air with a relative humidity of approximately 40% at normal room temperature and at 60 °C temperature with operation for 720 h. Liu et al. further employed Cu-phthalocyanine to act as HTL in combination with a printable carbon electrode [116]. This is a very good option for fabricating cheap scalable contact material. The attained PCE was 6.2% on an active area of 1 ${\mathrm{cm}}^{2}$ and 4.7% on an active area of 2.25 ${\mathrm{cm}}^{2}$.

**Figure 12:**
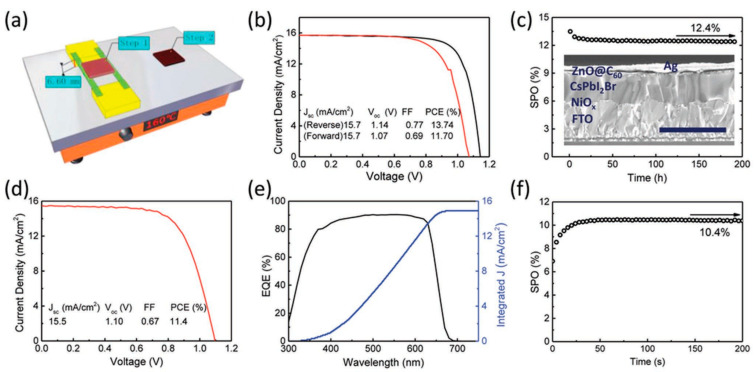
(**a**) Schematic illustration of InCl_3_:CsPbI_2_Br perovskite thin film fabrication via thermal radiation heating method (TRHM); (**b**) J-V characteristics of the champion PSC with an active area of 0.09 ${\mathrm{cm}}^{2}$; (**c**) SPO at 0.9 V forward bias as a function of time and the corresponding scanning electron microscope snapshot of the PSC with a 1 μm scale bar; (**d**) J-V characteristics of champion PSC with a larger active area of 1 ${\mathrm{cm}}^{2}$; (**e**) EQE spectra; and (**f**) SPO at 0.8 V forward bias as a function of time. Adapted with permission from [117].

Recently, Liu et al. demonstrated a thermal radiation heating method (TRHM), wherein the thermal treatment process can be performed without any contact [117]. They utilized two polyimide tapes to disconnect the layer of substrate from the hotplate, which permitted a uniform transition in phase from the edge of the substrate to the central region. The fabricated all-inorganic InCl_3_:CsPbI_2_Br perovskite film of 1 ${\mathrm{cm}}^{2}$ active area exhibited a much higher PCE of up to 11.4%, with a $J_{sc}\;$ of 15.5 mA cm^−2^, $V_{OC}\;$ of 1.10 V and an FF of 0.67. The SEM image and performance characteristics of this cell is shown in Figure 12.

The other major challenge pertaining to commercialization is the operational stability of the all-inorganic PSC during long runs. The operational stability of SCs means that they perform steadily and continuously under maximum power output in rain, thermal stress, oxygen ambience, day-night cycle, UV-light exposure and temperature variation [118,119]. The instability in PSCs is most probably engendered owing to perovskite film defects, such as imperfect interfacial charge transfer, vacancies, etc. [120,121]. These film defects induce phase segregation and ion migration, making the SC susceptible to external conditions and causing power output inconstancy. Moreover, the extensively utilized Spiro-OMeTAD also degrades the operational stability since the morphology of its film is unstable at high temperatures and due to the extremely hygroscopic nature of the lithium bis(trifluoromethanesulfonyl)imide LiTFSI dopant. In addition, metals such as silver and gold have the tendency to permeate inside the perovskite film or to become oxidized by iodine owing to the migration of iodine [122,123,124,125]. Hitherto, only a few investigations have been carried out that address the device stability issue in the presence of external environmental conditions. Most of the devices have been studied in the dark or under continuous illumination under an inert ambience. Despite these limitations, the findings on the stability of all-inorganic PSCs are promising. For instance, CsPbBr_3_ all-inorganic PSCs, having a configuration FTO/c-TiO_2_/m-TiO_2_/CsPbBr_3_/carbon without encapsulation, exhibited no PCE deterioration when stored under a relative humidity of 90–95% at ambient temperature even after three months [53]. They also showed thermal stability for 840 h at 100 °C. It is noteworthy that HTL and gold are fully substituted by carbon as a charge-collecting layer, thereby providing resistance against moisture. Nevertheless, the stability parameters still fall short against the requirements set by the International Electrotechnical Commission IEC61215:2016 standard of 1 000 h at 85% RH and 85 °C.

In order to ameliorate the operational stability, perovskite materials that are robust against external conditions, and the film deposition techniques, should be further developed to achieve steps towards commercialization. To this end, Jung et al. employed undoped stable poly (3-hexylthiophene) as HTL in organic–inorganic hybrid PSCs and fabricated high-performance perovskite SCs [126]. Future work will be focused on the optimization of the architecture. Regarding this, Arora et al. introduced a graphene layer between HTL and gold that probably prevented the permeation of gold nanoparticles into the perovskite layer [127,128].

## 6. Conclusions and Future Prospects

There is an urgent need for the development and commercialization of high-performance PSCs since fossil fuel is exhaustible while solar energy is renewable and eco-friendly. The SCs are required to be operated in open environments and hence need to be robust against external atmospheric conditions while exhibiting high performance and operational stability. Keeping this in mind, inorganic perovskite SCs should be preferred over organic–inorganic hybrid perovskite SCs since they offer high resistance against elevated temperatures. Although the maximum attained efficiency of inorganic hybrid PSCs is significantly lower than that of organic–inorganic hybrid PSCs, there are significant opportunities for their improvement, as discussed in this review. During the past five years, researchers have paid much attention to all-inorganic perovskite SCs, and their PCEs and operational stability have been significantly improved.

Inorganic perovskites with narrow bandgap are the best materials to attain high PCEs, but a major obstacle that remains is the phase instability of these perovskites under atmospheric conditions. The primary approach to a resolution will be to pursue compositional engineering since it significantly enhances the perovskite lattice entropy. In this review, I have attempted to survey the research on all-inorganic PSCs from their fabrication to commercialization. In addition, the compositional engineering of all-inorganic perovskite materials, the various film deposition techniques, and the mechanisms of energy loss have been discussed. Schemes to suppress energy losses and phase instability have also been presented.

The quality of perovskite films plays an important role in achieving high performance PSCs. In this regard, various methods have been successfully adopted by researchers to ameliorate film quality with ideal grain size and uniform coverage, including solution-processing and co-evaporation techniques. The crystallization dynamics are highly dependent on the deposition factors, such as evaporation rate, solvents and temperature, etc. Further research on the crystallization dynamics is crucial to properly tune the deposition parameters to achieve pinhole-free, smooth, and large grain-size films.

The Sn-based inorganic perovskites are the most extensively researched materials amongst the lead-free inorganic perovskites. Considering the Gold–Schmidt tolerance factor, the Sn-based inorganic perovskites must have a highly stable geometrical structure since Sn has a smaller radius compared to Pb. Nevertheless, the further development of these Sn perovskites is constrained because they are prone to rapid oxidation. Researchers have proposed methods to treat the perovskite surface with additives such as SnX_2_, which provides a shielding layer to the perovskite against atmospheric oxygen.

The uninterrupted and cheaper production of PSCs at large scale has been achieved using various printing technologies, such as roll-to-roll and slot-die, etc. These techniques have paved the way for the fabrication of flexible and portable electronics. Since the perovskite film deposition over large area substrates (upscaling) can deteriorate the performance of PSCs due to the challenges in controlling the homogeneity of the perovskite films, research in furthering strategies for modulating the morphology of substrates and solvent evaporation rate is very much needed. Since the PCEs of inorganic PSCs are improving and reaching an application feasibility level through continuous research, their long-term operational stability is likely to attract significant attention in the future. Moreover, attention should now be focused on the nucleation and growth mechanisms of the perovskite materials, which are mostly dependent on the deposition parameters.

## Figures and Tables

**Table 1:** Performance characteristics of various devices fabricated via two-step solution processing technique.

Device Configuration	$J_{sc}\;(mA\;cm^{-2})$	$\;\;\;\;\;V_{OC}\;\left(V\right)$	FF (%)	PCE (%)	Ref.
FTO/TiO_2_/CsPbBr_3_/PTAA/Au	6.7	1.25	73	6.2	[46]
FTO/TiO_2_/CsPb_0.97_Sm_0.03_Br_3_/PCBM/carbon	7.48	1.59	85.1	10.14	[47]
FTO/TiO_2_/GQDs/CsPbBr_3_/carbon	8.12	1.458	82.1	9.72	[48]
FTO/TiO_2_/GQDs&CISZ-QDs/CsPbBr_3_/carbon	7.35	1.522	84.3	9.40	[49]
FTO/TiO_2_/Cs_0.91_Rb_0.09_PbBr_3_/carbon	7.73	1.552	82.2	9.86	[50]
FTO/TiO_2_/CsPbBr_3_/PTAA/Au	6.24	1.28	74	5.95	[51]
FTO/TiO_2_/CsPbBr_3_/carbon	5.7	1.29	68	5.0	[52]
FTO/TiO_2/_CsPbBr_3_/carbon	7.4	1.24	73	6.7	[53]
FTO/TiO_2_/CsPbBr_3_/CdZnSe@ZnS-QDs/Spiro/Ag	7.25	1.498	79.6	8.65	[54]
FTO/TiO_2_/CsPbBr_3_/CsSnI_2_Br-QDs/carbon	8.7	1.39	75.5	9.31	[55]

**Table 2:** Lead-based inorganic perovskites SCs with different additives and their performance comparison.

$Additive{\;}^{*}$	Perovskite	$J_{sc}\;(mA\;cm^{-2})$	$\;\;\;\;\;V_{OC}\;\left(V\right)$	PCE (%)	SPO (%)	**Ref.**
HI	CsPbI_3_	12.00	0.80	2.90	1.70	[36]
EDAPbI_4_	CsPbI_3_	14.53	1.15	11.86	–	[57]
PTABr	CsPbI_3_	18.76	1.10	17.06	16.30	[58]
PVP	CsPbI_3_	14.88	1.11	10.74	–	[59]
PEAI	CsPbI_3_	16.59	1.07	12.40	–	[60]
DETAI_3_	CsPbI_3_	12.29	0.95	7.01	–	[61]
NDSB201	CsPb(I_0.98_Cl_0.02_)_3_	14.90	1.08	11.40	11.40	[62]
DMA	CsPbI_3_	16.65	0.99	12.62	–	[63]
DMSO	CsPbI_2_Br	15.33	1.22	14.78	14.67	[64]
Sn^2+^	CsPb_0.9_Sn_0.1_IBr_2_	14.30	1.26	11.33	–	[65]
Ge^2+^	CsPb_0.8_Ge_0.2_I_2_Br	12.15	1.27	10.80	–	[24]
Sr^2+^	CsPb_0.98_Sr_0.02_I_2_Br	15.30	1.04	11.30	10.80	[66]
Eu^2+^	CsPb_0.95_Eu_0.05_I_2_Br	14.63	1.22	13.71	13.34	[67]
Bi^3+^	CsPb_0.96_Bi_0.04_I_3_	18.76	0.97	13.21	13.17	[68]
Ca^2+^	CsPb_0.95_Ca_0.05_I_2_Br	17.90	0.945	13.50	13.30	[69]
Na^+^	Cs_0.94_Na_0.06_PbBr_3_	6.97	1.49	8.31	–	[50]
Li^+^	Cs_0.98_Li_0.02_PbBr_3_	6.95	1.45	7.87	–	[50]
K^+^	Cs_0.92_K_0.08_PbBr_3_	7.25	1.51	8.61	–	[50]
Rb^+^	Cs_0.91_Rb_0.09_PbBr_3_	7.73	1.55	9.86	–	[50]
K^+^	Cs_0.925_K_0.075_PbI_2_Br	11.60	1.18	10.00	9.00	[70]
Mn^2+^	CsPb_0.995_Mn_0.005_I_1.01_Br_1.99_	13.15	0.99	7.36	–	[71]
Sb^3+^	CsPb_0.96_Sb_0.04_I_3_	14.64	0.73	5.18	–	[72]
Sm^3+^	CsPb_0.97_Sm_0.03_Br_3_	7.48	1.59	10.14	–	[47]
Yb^3+^	CsPb_0.97_Yb_0.03_Br_3_	7.45	1.54	9.20	–	[47]
Er^3+^	CsPb_0.97_Er_0.03_Br_3_	7.46	1.56	9.66	–	[47]
Ho^3+^	CsPb_0.97_Ho_0.03_Br_3_	7.45	1.57	9.75	–	[47]
Tb^3+^	CsPb_0.97_Tb_0.03_Br_3_	7.47	1.59	10.06	–	[47]
	CsPbBr_3_	8.12	1.46	9.70	–	[48]

* EDA—ethylenediamine, PTABr—phenyltrimethylammonium bromide, PVP—poly-vinylpyrrolidone, PEAI—phenylethylammonium iodide, DETA—NH_3_^+^C_2_H_4_NH_2_^+^C_2_H_4_NH_3_^+^, NDSB201: 3-(1-pyridinio)-1-propanesulfonate DMA: dimethylammonium.

**Table 3:** Double-perovskites with their bandgaps and performance characteristics.

Double Perovskite	Bandgap [eV]	PCE [%]	*V_OC_* (V)	$J_{sc}\;(mA\;cm^{-2})$	FF	Ref.
Cs_2_SnI_6_	1.48	0.96	0.51	5.41	0.35	[90]
Cs_2_SnI_4_Br_2_	1.40	2.02	0.56	6.23	0.58	[90]
Cs_2_AgBiBr_6_	1.95, 2.20	2.23	1.01	3.19	0.69	[91,92]
Cs_2_TiBr_6_	1.80	3.28	1.02	5.69	0.56	[93]
Dy_2_NiMnO_6_	1.03	0.014	0.197	0.29	0.24	[94]
Lu_2_NiMnO_6_	0.98	0.00015	0.021	0.03	0.23	[94]
La_2_NiMnO_6_	1.40	0.17	0.336	1.85	0.27	[94]
Eu_2_NiMnO_6_	1.06	0.11	0.294	1.44	0.26	[94]

## Data Availability

Not applicable.
